# The Role of Gut-Derived Microbial Antigens on Liver Fibrosis Initiation and Progression

**DOI:** 10.3390/cells8111324

**Published:** 2019-10-27

**Authors:** Dishen Chen, Thanh H. Le, Haleh Shahidipour, Scott A. Read, Golo Ahlenstiel

**Affiliations:** 1 Storr Liver Centre, The Westmead Institute for Medical Research, University of Sydney, Westmead 2145, NSW, Australia; dche9355@uni.sydney.edu.au (D.C.); 19664268@student.westernsydney.edu.au (T.H.L.); haleh.shahidipour@health.nsw.gov.au (H.S.); 2 School of Medicine, Western Sydney University, Campbelltown 2560, NSW, Australia; 3 Blacktown Medical School, Western Sydney University, Blacktown 2148, NSW, Australia; 4 Blacktown Hospital, Blacktown 2148, NSW, Australia

**Keywords:** fibrosis, cirrhosis, alcoholic liver disease, NAFLD, NASH, intestinal permeability, bacterial translocation, innate immunity

## Abstract

Intestinal dysbiosis has recently become known as an important driver of gastrointestinal and liver disease. It remains poorly understood, however, how gastrointestinal microbes bypass the intestinal mucosa and enter systemic circulation to enact an inflammatory immune response. In the context of chronic liver disease (CLD), insults that drive hepatic inflammation and fibrogenesis (alcohol, fat) can drastically increase intestinal permeability, hence flooding the liver with gut-derived microbiota. Consequently, this may result in exacerbated liver inflammation and fibrosis through activation of liver-resident Kupffer and stellate cells by bacterial, viral, and fungal antigens transported to the liver via the portal vein. This review summarizes the current understanding of microbial translocation in CLD, the cell-specific hepatic response to intestinal antigens, and how this drives the development and progression of hepatic inflammation and fibrosis. Further, we reviewed current and future therapies targeting intestinal permeability and the associated, potentially harmful anti-microbial immune response with respect to their potential in terms of limiting the development and progression of liver fibrosis and end-stage cirrhosis.

## 1. Introduction

The progressive accumulation of extracellular matrix (ECM) in the liver, termed fibrosis, is the result of chronic liver damage due to a variety of insults: viral hepatitis (hepatitis B virus (HBV) and hepatitis C virus (HCV)), alcohol or fatty liver disease, drug-induced liver damage, or autoimmunity. While the prevalence of HCV- and HBV-mediated fibrosis has been on the decline since the advent of direct acting antivirals (DAAs) for HCV and improved vaccination/education strategies for HBV, other etiologies are on the rise [1]. The prevalence of non-alcoholic fatty liver disease (NAFLD) and its inflammatory form, non-alcoholic steatohepatitis (NASH), have increased in step with the obesity epidemic and are significant contributors to fibrosis, particularly in Western countries [1]. Indeed, while the prevalence of viral hepatitis dropped between 2000 and 2017, alcoholic cirrhosis increased by 16% and NASH cirrhosis by 33% [1].

The term gut–liver axis defines a bidirectional interaction between the gastrointestinal tract and the liver [2]. While the liver contributes bile acids, IgA, and antimicrobial peptides to the gut via the biliary tract, the portal vein transports gastrointestinal metabolites from the gut into the liver [2]. In the absence of disease, the mucosal barriers within the intestinal tract remain intact, preventing the transport of luminal microbes into the liver. In chronic liver disease (CLD) however, the intestinal barrier is impaired as a result of lifestyle choices (e.g., alcohol or obesity) or portal hypertension secondary to advanced fibrosis/cirrhosis, resulting in the translocation of microbes and their products into the blood.

Translocation of gut-derived antigens into the portal circulation enacts a potent inflammatory response in the liver, which has been well described in alcoholic and fatty liver disease, as well as liver cirrhosis [2]. These antigens not only drive hepatic inflammation and progressive fibrosis, but also contribute to mortality in end-stage liver disease due to their role in secondary infections such as spontaneous bacterial peritonitis (SBP) and hepatic encephalopathy [3]. While intestinal permeability is not the primary driver of liver inflammation and fibrosis, it has become evident that the inflammatory response to microbial antigens as a result of increased intestinal permeability strongly influences the progression of disease.

This review will focus on the multi-systemic nature of the gut–liver axis in health and disease. We have described (1) the intestinal barriers and mechanisms by which they become impaired in chronic liver disease, (2) the contribution of microbial antigens to liver inflammation and fibrosis, and (3), current therapies used to prevent either intestinal permeability or the hepatic inflammatory and fibrotic response.

## 2. Gut Microbiota and Bacterial Translocation in Chronic Liver Diseases

### 2.1. Gut Microbiota in Chronic Liver Diseases

The human gastrointestinal (GI) tract is estimated to contain more than 10^14^ microorganisms which collectively form the gut microbiota [4]. High motility and acidity within the esophagus and stomach limit colonization, however microbial numbers steadily increase throughout the small bowel, reaching the highest density in the colon where 10^12^ bacteria can be found in every gram of dry feces [5].

The intestinal microbiota is composed primarily of bacteria (60% of dry fecal mass), but is also abundant in archaea, eukarya, and viral species [6]. Sequencing of 16S ribosomal DNA from mucosal and stool samples has shown that Firmicutes and Bacteroidetes are the two most abundant phyla in human feces, followed by Proteobacteria, Actinobacteria, Fusobacteria, and Verrucomicrobia species [7]. Mucosal and fecal microbiota harbor distinct microbial profiles, characterized by an abundance of Bacteroidetes in stool, while human colonic crypts are colonized mainly with Firmicutes and Proteobacteria [8].

The non-bacterial intestinal microbiota contributes significantly to health and disease, but has been largely unappreciated to date. Eukaryotes account for less than 0.03% of fecal microbes and are primarily composed of 200–300 fungal species [9]. The study of intestinal viromes has been limited due to challenges in isolation and culture; however, recent metagenomic analyses have revealed that bacteriophages comprise ~90% of the gut virome and contribute significantly to bacterial dynamics [10].

The gut microbiota is essential for proper digestion and, furthermore, plays an important role in facilitating gut immune responses against potential pathogens. Indeed, commensal *Bacteroides* and *Lactobacillus* spp. can stimulate the release and activation of antimicrobial peptides such as C-type lectins and pro-defensins from intestinal Paneth cells [11,12], activate intestinal B cells to express secretory IgA [13,14], and stimulate the production of protective mucus from colonic goblet cells [15]—all mechanisms that prevent bacterial translocation across the mucosa [16]. Bacteriophage adherence to mucus layers has also been hypothesized to protect against bacterial colonization and infiltration [17].

Disturbances within the gut microbiota, termed “dysbiosis”, are linked to numerous diseases, many of which are hepatic in nature [18]. This is likely due to the bidirectional nature of the gut–liver axis: nutrient rich portal vein blood entering the liver originates from the gut, while hepatic bile from gallbladder travels into the intestines to facilitate digestion [19]. Consequently, the insults that drive CLD, including caloric excess (NAFLD/NASH), alcoholism (ALD), and biliary damage (primary sclerosing cholangitis, primary biliary cirrhosis), can have significant effects on the gut microbiota, leading to intestinal permeability and exacerbation of inflammation and fibrosis.

Many studies have suggested an association between gut microbiome alteration and chronic liver diseases. Both Mouzaki et al. and Silva et al. demonstrated a reduction in *Bacteroidetes* and *Firmicutes* spp. in both NAFLD and NASH patients compared to adult healthy controls [20,21]. In pediatric studies, Zhu et al. measured a decrease in Firmicutes and increased Bacteroidetes in children with obesity or NASH [22]. A more recent, larger cohort study challenged these findings, finding a decrease of total Bacteroidetes in both NASH and NALFD pediatric patients, in agreement with adult studies [23].

In ALD, a reduction of *Lactobacillus* spp. has been recorded in both alcohol-consuming patients and mouse models [24,25]. Lactobacilli are beneficial bacteria commonly used in probiotics that have been shown to inhibit pathogen colonization [26]. Patients with ALD have also been found to have lower abundance of Bifidobacterium and Enterobacterium, and increased Proteobacteria, Fusobacteria, and Actinobacteria [27,28].

Changes in patient gut microbiota have also been measured in the context of worsening disease state. Indeed, significant differences in gut microbiota have been observed in NALFD subjects who had progressed to steatohepatitis or moderate fibrosis (F ≥ 2) when compared to patients with earlier stages of the disease. Boursier et al. found that NASH patients possessed a significantly larger abundance of *Bacteroides* and a reduction in *Prevotella* compared to NAFLD patients [29]. Recently, Bastian et al. also confirmed a significantly higher proportion of *Bacteroides* in fibrotic (F2–F4) patients compared to patients with minimal fibrosis (F0–F1). Two large studies by Loomba et al. and Caussy et al. also found a reduction in *Firmicutes* spp. and an enrichment of *Proteobacteria* spp. in patients with cirrhosis compared to those with minimal fibrosis [30]. In addition, Bajaj et al. recently demonstrated that periodontal therapy improves gut dysbiosis and systemic inflammation in cirrhotic patients [31]. Cirrhotic patients treated with scaling and root planning followed by oral hygiene showed a reduction in Enterobacteriaceae and Streptococcaceae, and a decrease of inflammatory markers interleukin (IL)-1β and IL-6 [31]. Together, these findings suggest that certain bacteria, likely Bacteriodes and *Proteobacteria* spp., and other factors such as oral health may play important roles in liver fibrosis progression.

### 2.2. Physical and Chemical Barriers of the Intestinal Mucosa

**Figure 1:**
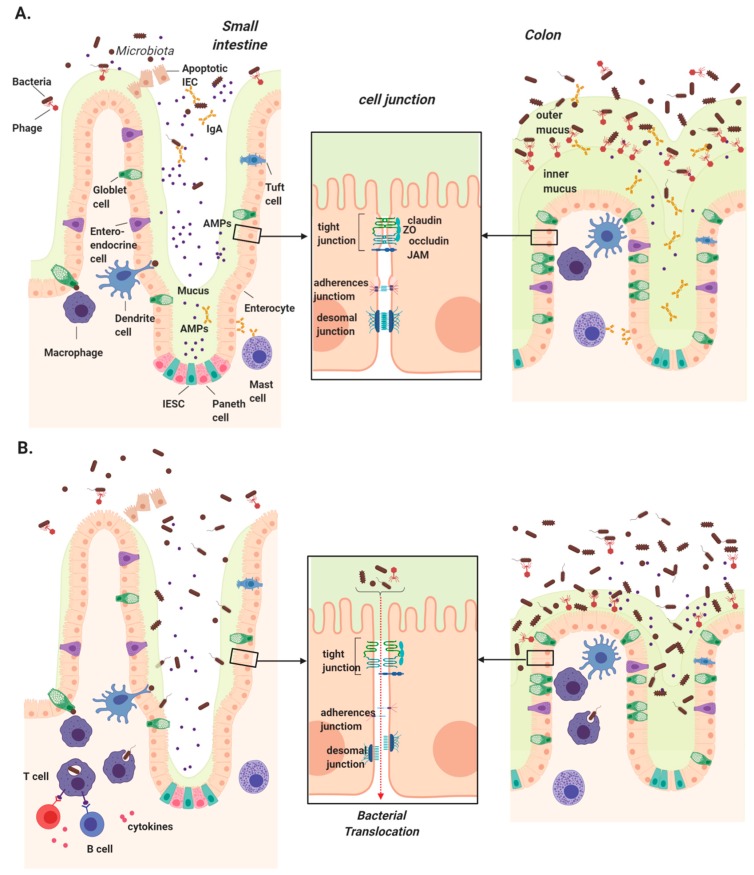
Intestinal mucosal barriers in health and chronic liver disease. (**A**) Several physical and chemical defenses make up the intestinal mucosa, which serves to protect us from luminal microbes. Intestinal epithelial stem cells (IESCs) located at the base of crypts give rise to all epithelial cells. Goblet cells secrete mucins to form a thin mucus layer in the small intestine and two thick layers in colon, the innermost of which is devoid of bacteria. Enterocytes/colonocytes and Paneth cells secrete antimicrobial peptides (AMPs) primarily in the small intestine, while mast cells secrete IgA, which travels through the epithelium and is concentrated in the colon. Underlying the epithelium, dendritic cells and macrophages continuously surveil luminal contents using trans-epithelial dendrites. (**B**) Disruption of these physical barriers can lead to intestinal permeability and increased microbial translocation in chronic liver disease. These include the reduction of secreted mucus, AMPs, and IgA, permitting microbial access to the epithelial layer. Downregulation, altered localization, or rearrangement of tight junction components can also significantly impact intestinal permeability, allowing microbial translocation into the portal circulation where they are transported into the liver.

To maintain a healthy coexistence with commensal microbes and prevent bacterial dissemination, the gastrointestinal tract is lined by a cellular epithelium. This physical barrier is composed primarily of epithelial cells, with the addition of specialized cell types that differ between the small and large intestine. While all epithelial cells arise from intestinal epithelial stem cells (IESC) at the base of crypts, they differentiate into a variety of cells, including enterocytes (colonocytes in the large intestine), goblet cells, Paneth cells, tuft cells, and Microfold cells (M cells) [32]. Apart from hormone-secreting enteroendocrine cells and nutrient-absorbing enterocytes, the remaining epithelial cells are largely responsible for defending against microbial invasion (Figure 1).

Goblet cells secret mucin proteins to form a highly glycosylated mucus layer over the vast epithelial surface, and this layer is significantly thinner in the small intestine compared to the colon due to a lower goblet cell density and bacterial colonization [33]. IgA secreted across the intestinal epithelium also comprises a significant component of the chemical defense. IgA is secreted by plasma cells in lymphoid follicles of the lamina propria, and transported via polymeric immunoglobulin receptors (pIgR) on the basolateral surface of epithelial cells into the lumen [34].

Epithelial integrity is maintained by junctions between intestinal epithelial cells (IECs) that provide selective nutrient permeability while preventing microbial translocation. There are three major types of cell junction that typically form near the apical end of the cells’ side walls: tight junctions (TJs), gap junctions, and adherens junctions [35]. Among them, TJs form the most rigid and impenetrable seal, hence their name. This junction is a complex of more than 50 proteins, most of which are transmembrane proteins such as occludin, claudin, and the junctional adhesion molecule (JAM) family proteins such as zonula occludens (ZO)-1, which connect with the cytoskeleton and form fibrils with adjacent cells [36,37].

### 2.3. Bacterial Translocation in Chronic Liver Disease

The intestinal mucosa represents the barrier protecting against luminal microbes while allowing the selective passage of digested nutrients into the circulation. This property, termed intestinal permeability, allows the intestinal tract to fulfill its absorptive function via two pathways [38]. The transcellular pathway enables macromolecules such as glucose, amino acids, or bacterial antigens to move through cells via enterocyte, M cell, and goblet cell transporter-mediated transcytosis or endocytosis. The paracellular pathway allows water and minerals to diffuse through the interspace between adjacent epithelial cells [39]. Passive transport in intestinal epithelium is, however, tightly controlled by the proteins making up the cell TJs [40]. Currently, intestinal permeability can be measured using in vivo or in vitro functional assays [39]. In vivo assays evaluate urinary or blood non-metabolized sugar such as lactulose/mannitol, polyethylene glycols (PEGs), Cr-labeled ethylenediaminetetraacetic acid (^51^Cr-EDTA), ovalbumin (OVA), or Fluorescein isothiocyanate dextran (FITC-dextran) following ingestion. In vitro assays indirectly measure intestinal permeability by detecting biomarkers such as bacteria, lipopolysaccharides (LPS), endotoxin antibodies, or bacterial fermentation products in lymph/blood or by histologically examining TJ protein localization and expression.

Even in the absence of disease, bacteria can be transported across the intestinal epithelium into the lamina propria where they can interact with mesenteric lymph nodes (MLN) and extra-intestinal organs via the blood [41]. This process was termed bacterial translocation by Berg and Garlington in 1979, and has since been expanded to include other luminal microbes such as viruses and fungi [42]. In fact, sequencing and culture methods have detected low levels of viable and non-viable microbes, microbial DNA, and antigens in the MLN as well as in other “sterile” organs, including the liver [43,44]. Bacteriophages have also been detected in various sites within the human body including the blood, kidney, and liver [45], however their immunogenicity remains uncertain.

Increased bacterial translocation (BT) is associated with gastrointestinal diseases and the extra-intestinal conditions of the liver, kidney, and brain, among others [46,47,48]. As early as 1995, Berg et al. identified three factors that contribute to BT: bacterial growth or dysbiosis, intestinal permeability, and immune deficiency [41]. These factors have been identified in various forms of chronic liver disease, and are outlined below and in Table 1.

#### 2.3.1. Alcoholic Liver Disease

In alcoholic fatty liver disease, viable bacteria, endotoxin, and LPS have been observed in the blood of both animal models and ALD patients, for which there are many potential mechanisms [49,50]. Small intestinal bacterial overgrowth (SIBO) in chronic alcoholics is more prevalent than healthy controls [51], perhaps due to extended orocaecal transit time in alcoholics compared to social drinkers [52]. SIBO has been suggested to mediate translocation due to differences in mucosal defense in the small intestine, primarily a reduction in mucus secretion. Increased intestinal permeability has also been reported in alcoholic cirrhosis, ALD, and even in non-cirrhotic alcoholics using in vivo assays [53,54,55].

In human studies, a reduction in the number of small intestinal villi, goblet cells, and TJ protein ZO-1 expression in the colon were observed in chronic alcoholics [56,57,58]. Furthermore, cell culture and animal studies suggest that ethanol and its metabolite, acetaldehyde, can alter intestinal barrier function by (1) inducing epithelial cell apoptosis, (2) disrupting TJs by downregulating ZO-1, occludin, and claudin and redistributing ZO-1 into cytoplasm, and (3) displacing the cytoplasmic skeleton [59,60,61,62].

Lastly, the production of intestinal IgA and the quantity of immune cells have been shown to be significantly altered in cases of chronic alcohol consumption and in ALD patients. While systematic IgA is increased in alcoholic liver diseases due to intestinal permeability, fecal IgA and IgA-producing B cells within the lamina propria are reduced in animal models of ALD [63,64]. Recently, a significant decrease in the number and activation state of mucosa-associated invariant T (MAIT) cells, a key component in antibacterial immune defense, was shown in ALD patients [65].

#### 2.3.2. Non-Alcoholic Fatty Liver Disease

Multiple-pathogen-associated molecular patterns (PAMPs) such as peptidoglycan, LPS, and bacterial DNA have been detected in NALFD/NASH patient blood, and are linked to metabolic syndromes and obesity [66,67,68]. Two key drivers of bacterial translocation in NAFLD are SIBO and intestinal permeability, having been documented in numerous studies [69]. Increased intestinal permeability has been documented in both adult [70] and pediatric [71] NAFLD studies, as measured by 51Cr-EDTA and lactulose–mannitol assays, respectively. Moreover, intestinal permeability was associated with the severity of inflammation and fibrosis in children [71]. Clinical studies by Miele et al. and later by Xin et al. revealed that deregulation of TJ proteins may be responsible for intestinal permeability, demonstrating a reduction of ZO-1 and occludin expression in parallel with increased intestinal permeability in NAFLD patients [70,72]. Both in vitro and animal model studies of obesity further suggest that bile acids and leptin can stimulate intestinal permeability [73,74].

There is also an indication that liver damage as a result of NASH can directly contribute to loss of barrier integrity [67]. A meta-analysis performed by Luther et al. found a higher rate of intestinal permeability in NASH patients compared to NAFLD alone. A further study using a mouse model of NASH indicated that intestinal permeability occurs only after initial liver injury and the induction of pro-inflammatory cytokines [67].

Gut immune alteration is also a factor that can contribute to enhanced bacterial translocation in NAFLD. Luminal IgA and IgA-positive cells within ileal and colonic tissue are decreased in mouse models of NASH fed the methionine/choline-deficient diet [75]. Collective studies of innate and adaptive immunity on animals and patients with obesity have reported an increase in inflammatory cytokine expression and pro-inflammatory cluster differentiation (CD)4^+^ and CD8^+^ T cell, but an opposite trend in regulatory T cells [76]. These pro-inflammatory cytokines, such as interferon (IFN)-γ, IFN-α, and IL-6, have been shown to disturb intestinal TJs, allowing the translocation of luminal antigens across the intestinal barrier [77].

#### 2.3.3. Liver Cirrhosis

Due to clinical associations with bacterial infection, microbial translocation is often examined in the context of liver cirrhosis. BT in cirrhosis has been identified using such methods as lymph node homogenate bacterial culture and bacterial DNA sequencing in cirrhotic patient blood [78,79]. Importantly, translocated bacteria, dominated by the Proteobacteria phylum, are abundant in the portal vein, as well as the hepatic and peripheral blood of decompensated cirrhotic patients [80]. When compared to healthy controls, SIBO is also significantly more common in patients with cirrhosis, particularly following decompensation [81,82]. Portal hypertension and abnormal small bowel motility are likely related to prevalent SIBO in decompensated cirrhosis [83].

Intestinal permeability as assessed by dual-sugar ingestion assays has been found to increase in both the small and large intestine of patients with decompensated/advanced cirrhosis [84,85,86]. Reduction in the expression of TJ proteins occludin and claudin-1 in cirrhotic patients may provide a mechanism for this increased permeability [87]. Nonetheless, electron microscopy experiments performed by Such et al. ten years prior demonstrated intact TJs in the duodenal epithelium of cirrhotic patients, but enlarged interspace between enterocytes [88]. More recently, an examination of cirrhotic mice treated with carbon tetrachloride (CCL_4_) showed a reduction of mucin (MUC)2 expression, mucus thickness, and goblet cell number, as well as an increase in intestinal permeability associated with bacterial overgrowth and translocation. The authors also suggested a modulatory role of the bile acid receptor Farnesoid X receptor (FXR), due to the restoration of TJ protein expression, goblet cell number, and bacterial translocation following FXR agonist treatment [89].

Although clinical associations have yet to be found, alterations in intestinal humoral and cellular immunity within gut-associated lymphoid tissues (GALT) have also been observed in cirrhotic models. An increase in bacterial translocation in cirrhotic rat models can activate monocytes and dendritic cells in GALT, releasing pro-inflammatory cytokines such as tumour necrosis factor (TNF)-α and IL-12 [90,91] that have been shown to increase intestinal permeability by deregulating ZO-1 and claudin 1 within the TJs [92,93]. While dendritic cells have been shown to open TJs to allow microbial sampling of the lumen, an increased incidence of DC sampling has been measured in cirrhotic rats, and is associated with increased translocation of bacterial DNA [91].

Studies in recent years have provided sufficient evidence to suggest that microbial translocation can drive the development and exacerbation of chronic liver diseases. However, the detailed cellular and mechanisms implied by this association will need more efforts to elucidate.

## 3. Hepatic Recognition of Microbial Ligands: The Role of Pattern Recognition Receptors

Pattern recognition receptors (PRRs) are a group of host sensors that recognize antigens derived from both foreign and endogenous sources. PRRs are essential initiators of the inflammatory and immune responses that defend against foreign microbial invaders as well as endogenous cellular debris known as damage-associated molecular patterns (DAMPs). While these sensors are an essential component of hepatic immunity, they can contribute to chronic inflammation and fibrosis progression in response to prolonged activation. As outlined in the previous section, the intestinal barrier can become impaired in CLD, allowing continuous translocation of microbial antigens into the portal circulation. This section aimed to characterize the hepatic response to these antigens as it relates to inflammation and fibrosis.

The cellular drivers of liver fibrogenesis are the myofibroblast-like hepatic stellate cells (HSCs). They are largely responsible for wound healing in their steady state, but drive fibrogenesis through ECM deposition upon chronic activation [95]. HSC fibrogenesis can be triggered either directly, via induction of PRRs on HSCs, or indirectly via inflammatory signals produced by neighboring cells such as hepatocytes and Kupffer cells (KCs) [95]. This section covers PRRs that contribute to fibrosis development, with in vivo evidence of inflammatory and fibrogenic activity in CLDs, including toll-like receptor (TLR) 2, TLR3, TLR4, TLR5, TLR7 TLR9, nucleotide-binding and oligomerization domain (NOD)-like receptors (NLRs), c-type lectin receptors (CLRs), and stimulator of interferon genes (STING). General information regarding PRRs in CLD is summarized in Table 2.

### 3.1. Toll-Like Receptors

#### 3.1.1. TLR2

Hepatic upregulation of TLR2 has been observed in patients with NAFLD/NASH [98,100] and fibrosis due to chronic viral infection [152]. In contrast, ALD patients show significantly lower expression of hepatic TLR2 compared to healthy controls [101].

In murine models of CLD, TLR2 significantly contributes to hepatic inflammation and fibrosis. In chronic ethanol-binge-fed mice, TLR2 is crucial for hepatic IL-1β, IL-6, and TNF-α-related liver injury and inflammation, as well as neutrophil-mediated hepatic injury [102,103]. In addition, using the choline-deficient L-amino-defined (CDAA)-diet-induced NASH model, TLR2 deficiency improved hepatic inflammation and injury by reducing Kupffer cell inflammasome activation and pro-inflammatory cytokine production, suggesting a KC-dependent inflammatory pathway mediated by TLR2 [96]. In contrast, TLR2 was protective in the NASH methionine/choline-deficient (MCD) diet model, as demonstrated by an increase in ALT and TNF-α in TLR2 KO mice [104].

In mouse models of fibrosis, TLR2 was reported to have limited contribution to fibrogenesis in bile duct ligation (BDL) mice [105]. In contrast, TLR2^−/−^ mice treated with CCL_4_, possessed significantly impaired HSC activation with reduced collagen deposition, pro-inflammatory cytokine and α-smooth muscle actin (SMA) expression [107]. In addition, they demonstrated attenuated mitogen-activated protein kinase (MAPK) and nuclear factor kappa-light-chain-enhancer of activated B cells (NF-κB) activation compared to wild type (WT) fibrotic mice. A neutrophil-driven mechanism of fibrogenesis in CCL_4_-induced fibrosis has also been attributed to TLR2-mediated hepatic chemokine C–C motif ligand (CXCL)2 production [106]. Lastly, TLR2 knockout (KO) in CDAA-diet-induced NASH mice significantly dampened HSC activation, collagen deposition, α-SMA expression, and transforming growth factor (TGF)-β expression, thus, ameliorating NASH-associated fibrogenesis [96].

#### 3.1.2. TLR3

The protective and anti-inflammatory role of TLR3 has been reported in mice fed with high-fat diet (HFD) followed by binge drinking to induce liver injury. Stimulating TLR3 using poly I:C resulted in elevated HSC and KC IL-10 expression, as well as reduced hepatic expression of TNF-α, IL-6, CXCL2 and impaired liver injury [113].

TLR3 signaling is well characterized in murine natural killer (NK) cells, where activation of TLR3 results in a potent anti-fibrotic effect in both 3,5-diethoxycarbonyl-1,4-dihydrocollidine (DDC)-diet- and CCl_4_-induced murine fibrosis models [153,154]. TLR3 has been shown to work synergistically with IL-18 to activate the p38/PI3K/Akt pathway, thus stimulating NK cells to kill HSCs via TNF-related apoptosis-inducing ligand (TRAIL)-mediated degranulation [114]. In contrast, TLR3 is pro-fibrotic in the CCL_4_ fibrosis model, where TLR3 KO results in downregulation of IL-6, TNF-α, and pro-fibrotic markers [115]. Interestingly, the authors concluded that CCL_4_-treated hepatocyte exosomes stimulated HSC TLR3 signaling to drive γδ T cell IL-17 production and fibrosis progression [115].

#### 3.1.3. TLR4

TLR4 is the most thoroughly studied PRR in the setting of CLD. Hepatic and serum TLR4 is significantly upregulated in NASH patients, with elevated levels of circulating LPS in peripheral [100,116] and portal vein blood [155]. High serum levels of TLR4 have also been proposed as a predictive non-invasive marker for liver fibrosis development in NASH patients [156]. Although hepatic expression of TLR4 has not been studied in patients with ALD, peripheral blood mononuclear cells (PBMCs) from patients with ALD showed sensitized responses towards LPS treatment [117].

The role of TLR4 in murine models of liver inflammation has been well studied. TLR4-dependent ROS production and TLR4-dependent interferon regulatory factor (IRF)3 activation in the liver are required to drive hepatic inflammation in mice with alcoholic hepatitis [120]. A similar study showed that hepatic inflammatory cytokines were significantly downregulated in hepatocyte-selective-TLR4-deficient mice fed with a liquid diet containing 5% ethanol [119]. The importance of TLR4 in NASH development was further emphasized in a murine NASH model using high-fat, high-cholesterol (HFHC)-diet fed ApoE KO mice, showing a TLR4-mediated ROS production and triggering pro-inflammatory cytokine expression in KC [122]. Linking TLR4 to NAFLD pathogenesis, fatty acids such as palmitate can also trigger ROS production in a TLR4-dependent manner, inducing IL-1β and TNF-α production from liver macrophages [121].

TLR4-mediated fibrosis has been interrogated in a variety of mouse models. In BDL mice, the TLR4–MyD88–NF-κB pathway in HSCs has been shown to upregulate pro-inflammatory cytokine production, α-SMA, TIMP1, and TGF-β expression, and ECM deposition [105,123]. In addition, TLR4-mediated downregulation of Bambi (a TGFβ pseudoreceptor) was shown to sensitize quiescent HSCs for subsequent activation [105,123]. Using the transforming growth factor beta-activated kinase 1 (TAK1) KO murine model of fibrosis [124], TLR4 and MyD88 double KO mice also demonstrated reduced α-SMA, TIMP1, and TGFβ expression and collagen deposition, supporting the involvement of TLR4–MyD88–NF-κB signaling in hepatic fibrogenesis [124].

#### 3.1.4. TLR5

Peritoneal injection with the TLR5 ligand flagellin has been shown to induce significant TLR5-mediated liver injury in mice, resulting in IL-6, IL-8, and IL-1β production, coupled with neutrophil and macrophage infiltration into the liver [126]. In the context of NASH, however, hepatocyte TLR5 may possess a protective effect. In mice fed with MCD diet to induce NASH, selective hepatocyte deficiency of TLR5 was shown to exacerbate liver inflammation and fibrosis via elevated expression of TNF-α, monocyte chemoattractant protein (MCP)1, and IL-1β, as well as Timp1, Mmp9, Col1, and collagen deposition [125]. These conflicting results may, however, be the result of hepatocyte versus whole-body knockout of TLR5 and require further study.

Unlike hepatocyte-specific TLR5 KO, whole-body KO ameliorates inflammation and fibrogenesis in the CCL_4_ fibrosis model. TLR5 KO mice demonstrated a significant reduction of inflammatory mediators TNF-α, IL-6, and IL-1β and fibrogenesis, as indicated by downregulation in α-SMA, TGF-β, TIMP1, and collagen deposition compared to WT mice [127]. A significant reduction in NF-κB and MAPK signaling activity was measured in activated HSCs, suggesting a mechanism of hepatic fibrogenesis mediated by the TLR5-activated NF-κB/MAPK signaling pathway in HSCs [127].

#### 3.1.5. TLR7

The role of TLR7 in NASH/NAFLD, liver fibrosis, or liver cirrhosis has been widely overlooked in the clinical setting; however, recent studies suggest that ALD-mediated inflammation and fibrosis is linked to hepatic TLR7 overexpression [130,131]. Moreover, TLR7-mediated IFN production is stimulated by alcohol in primary human hepatocytes and is correlated with patients with more advanced fibrosis as well as higher expression of fibrotic markers α-SMA, collagen I, and Timp1 [130].

The role of TLR7 in ALD, NASH/NAFLD, and fibrosis development has been established primarily in mouse models. A recent study using ethanol (25% w/v) feeding to stimulate alcoholic hepatitis showed that activation of TLR7 significantly upregulated expression of pro-inflammatory cytokines and the endogenous TLR-7 agonist let-7b from hepatocytes, hence exacerbating hepatic inflammation [131]. Roh et al. recently showed that TLR7 deficiency significantly reduced the degree of hepatic steatosis and inflammation in a MCD-diet-induced NASH mouse model, examined by H&E staining, as well as TNF-α and IFN-α production from KC and hepatic dendritic cells, respectively [132]. In contrast, TLR7 has been identified as a protective factor in hepatic fibrosis development in both CCL_4_ and BDL murine fibrosis models. TLR7 KO mice expressed higher levels of hepatic pro-inflammatory cytokine and fibrosis marker expression as well as exacerbated collagen deposition [129]. Moreover, dendritic cell expressed type I IFNs upon TLR7 stimulation, triggered KC IL-1 receptor antagonist expression and ultimately suppressing IL-1-dependent liver injury and inflammation [129].

#### 3.1.6. TLR9

In humans, hepatic TLR9 expression is upregulated in NASH patients [100,133], while high serum levels of bacterial DNA (TLR9 ligand) have also been linked with liver cirrhosis and liver fibrosis [134,135]. Indeed, an increase in circulating bacterial DNA has been measured in patients with alcoholic and fatty liver disease prior to fibrotic development [136]. In addition, acute binge drinking has been shown to significantly increase serum bacterial DNA and pro-inflammatory cytokines such as IL-6, TNF-α, and IL-1β to indirectly contribute to liver fibrosis [135,139,157].

The role of TLR9 in chronic liver inflammation has been well established in animal models. In mouse models of CDAA-induced NASH, activation of TLR9 can stimulate IL-1β expression in KCs, mediating steatohepatitis and hepatocyte apoptosis driven by lipid accumulation [137,138]. In addition, the pro-inflammatory role of TLR9 in NASH has been further confirmed in atherogenic diet-fed NASH and *foz* mouse models, demonstrating reduced pro-inflammatory cytokine production and attenuated hepatic neutrophil infiltration in TLR9^−/−^ mice [133]. Similarly, evidence for TLR9-mediated liver injury and inflammation in chronic-binge-ethanol-fed mice was shown to be driven by IL-1β expression as well as TLR9/TLR2-dependent hepatic neutrophil infiltration mediated by CXCL1/2/5 expressed from hepatocytes and HSC [102,139].

Despite the inflammatory role of TLR9 in human NASH, TLR9 KO failed to improve hepatic fibrosis in murine NASH models [133]. TLR9 does, however, seem to influence fibrosis development in other models of fibrosis including murine BDL, where TLR9^−/−^ mice demonstrated significantly alleviated fibrogenesis and HSC activation compared to WT mice [140]. Similar results were found using the spontaneous fibrosis Tak1ΔHep mouse model, also demonstrating a reduction in liver inflammation and fibrosis in TLR9 KO mice [124]. Moreover, stimulation of TLR9 using CpG DNA directly activated the fibrogenic phenotype in primary mouse HSCs and the immortalized human HSC LX-2 cell line [158].

### 3.2. NOD-Like Receptors

The influence of NLRs on liver inflammation and fibrosis was thoroughly reviewed recently by Xu et al. [145]. NOD1, NOD2, and nucleotide-binding oligomerization domain, leucine-rich repeat- and pyrin-domain-containing (NLRP)3 are the main NLRs driving hepatic inflammation, liver injury, and liver fibrogenesis. NOD1-mediated neutrophil recruitment has been described following acute liver injury and inflammation in a CCL_4_-treated murine model [159]. In addition, activation of NOD-2 by muramyl dipeptide, a bacterial peptidoglycan motif, can induce NF-kB-dependent hepatic expression of pro-inflammatory cytokines to indirectly orchestrate liver inflammation and fibrosis [145,160]. Furthermore, it has been demonstrated that NLRP3–inflammasome pathway is activated in murine NASH to induce hepatic TNF-a, IL-6, and IL-8 production [144,145].

Hepatocyte stimulation of NOD1 via its ligands can activate the NF-kB and MAPK pathway to induce CXCL1 and CCL5 production, promoting wound healing and fibrogenesis [161]. NLRP3–inflammasome has been demonstrated to be a direct contributor in hepatic fibrogenesis. Activation of NLRP3 undoubtedly triggers direct activation of HSC to enhance matrix deposition, TGF-β expression, and fibrosis progression [142].

### 3.3. Anti-Fungal PRRs and Liver Fibrosis

The role of the CLR dectin-1 in liver inflammation and fibrosis development has been thoroughly studied. Ethanol-containing-diet-fed mice were found to have elevated serum β-glucan level and hepatic injury, which was significantly reduced upon treatment with anti-fungal agent [147]. More importantly, plasma β-glucans enhanced IL-1β expression in KC to drive hepatic inflammation that was absent in dectin-1 deficient mice [147]. A further study showed that hepatic expression of dectin-1 was upregulated in a thioacetamide (TAA)/CCL_4_ fibrosis mouse model, and that dectin-1 negatively regulated the expression of TLR4 and its co-receptor CD14 to mitigate fibrosis development and hepatic inflammation [146]. Knocking out dectin-1 exacerbated liver fibrosis and inflammation, as demonstrated by increased expression of TNF-α, IL-6, and MCP-1, neutrophil and macrophage influx, and fibrosis progression [146].

### 3.4. STING

In view of its recent discovery, there have been a limited number of human studies exploring the role of STING in CLD. Luo et al. reported an overexpression of STING in the non-parenchymal cells within the liver tissue of NAFLD patients, albeit with no examination of their relationship to disease activity [148]. By utilizing the alcohol-fed mouse ALD model, Petrasek et al. demonstrated that activation of the STING–IRF3 pathway stimulates pro-inflammatory cytokine production in the liver [149]. In the same study, alcohol-induced liver injury was also shown to trigger the STING–IRF3 pathway by endoplasmic reticulum (ER) stress, promoting the mitochondrial apoptotic pathway in hepatocytes [149]. Knocking out STING in HFD-induced NAFLD and MCD-induced NASH murine models attenuated hepatic activation of IRF3 and NF-κB pathways, and significantly downregulated expression of pro-inflammatory cytokines to alleviate NAFLD/NASH severity [148]. Similar findings were reported by Yu et al. using HFD- and MCD-diet-induced NASH mice; hepatocytes releasing mitochondrial DNA during NASH development led to the activation of STING–IRF3 pathway on KC to trigger pro-inflammatory cytokine production and hepatic inflammation in NASH [150].

Taken together, the above studies suggest that STING plays an important role in both hepatic inflammation and hepatic fibrosis in NASH [148,150]. Similar findings were elucidated in a CCL_4_-induced liver fibrosis murine model by Iracheta-Vellve et al. Hepatocytes were shown to undergo significant ER stress resulting in STING–IRF3 activation and induction of the mitochondria-dependent apoptosis pathway. Hepatocyte cell death was shown to activate HSC expression of α-SMA and COL1A2, collagen deposition, and fibrosis progression [151].

## 4. Therapies to Prevent Microbe-Driven Liver Inflammation and Fibrosis

The primary interventions for alcoholic and fatty liver disease have focused on lifestyle modifications: abstaining from alcohol and improving patient diet and exercise regimens. In more severe cases of obesity, surgical interventions (e.g., bariatric surgery) are required in combination with significant lifestyle adjustments. Unfortunately, even in the case of surgical interventions, patient compliance towards dietary and alcohol restrictions is often lacking, rendering the development of novel therapeutics a top priority.

In cirrhotic patients, dietary interventions and abstinence from alcohol drastically improve survival [162]. These patients also generally require treatment of portal hypertension, which is the contributing factor to intestinal permeability. Unfortunately, however, in decompensated cirrhosis, treatment of portal hypertension using beta-blockers and anti-hypertensives can dangerously reduce mean arterial pressure [163]. In addition, there is an increased risk of susceptibility to infections due to poor phagocytic capacity in the liver and increased likelihood of ascites: the accumulation of fluid in the peritoneal cavity [3]. These aspects further underline the vital need for novel therapies to prevent the progression of fibrosis, particularly in end-stage liver disease where there is currently a lack of effective treatment. Limiting the exacerbation of inflammation and fibrosis by microbial translocation into the liver is therefore an avenue that must be explored. We aimed to summarize current and potential therapies directed at (1) reducing microbial translocation, and (2) limiting the harmful response to microbial antigens in the liver.

### 4.1. Therapies to Reduce Intestinal Permeability

Studies examining intestinal permeability in ALD [24,53,54], NAFLD/NASH [67,70,164], and fibrotic liver disease/cirrhosis [86,87,165] have focused primarily on the reduction in TJ proteins such as ZO-1 and Claudin-1 [70,87,94], though the mechanisms by which this loss occurs remains largely unknown. Loss of intestinal mucus or mucosal IgA production [166] can also significantly increase intestinal permeability; however, these mechanisms have been largely overlooked in CLD.

Expansion of pathogenic bacterial species both within the colon and into the SIBO have been documented in CLD [70,167], motivating the examination of antibiotics and prebiotics as potential therapies. Antibiotic studies commonly use a broad spectrum and poorly absorbed antibiotics such as neomycin or rifaximin to achieve a gut-targeted intestinal decontamination. In rodent models of obesity [168], long-term ethanol exposure [169], NASH [170], and fibrosis [105], antibiotic treatment has been shown to reduce intestinal permeability and subsequent liver injury. In humans, a number of clinical trials are underway to assess the efficacy of antibiotics for the treatment of AH, NASH, and cirrhosis (reviewed in [171]). Early data suggests that short-term rifaximin treatment can reduce serum endotoxin and liver inflammation in NAFLD/NASH patients [172], whereas rifamixin prophylaxis over 24 weeks can significantly reduce hospitalization and mortality among cirrhotic patients [173]. These data support previous findings that rifaximin can reduce the risk of complications associated with cirrhosis, including hepatic encephalopathy, variceal bleeding, and SBP [174]. Importantly, antibiotics such as levofloxacin and metronidazole, but not rifaximin, can significantly increase gut proteases, thus contributing to intestinal permeability in both humans [175] and rats [176]. An antibiotic-mediated reduction in anti-proteolytic bacterial richness and abundance in the colon is thought to contribute to gut permeability.

Significant microbial perturbations caused by broad spectrum antibiotics have stimulated interest in probiotics (beneficial microbes) and prebiotics (beneficial microbial substrates) for the improvement of intestinal health. In mouse models of ethanol-induced liver injury, probiotic *Lactobacillus rhamnosus* strains have been shown to reduce serum endotoxin, hepatic oxidative stress, and inflammatory TNFα production [177,178]. While intestinal permeability was not assessed in these studies, Wang et al. demonstrated that *Lactobacillus* probiotics could prevent the alcohol-induced loss of intestinal ZO-1, Caludin-1, and Occludin-1, keeping the intestinal epithelium intact [179]. In rats, a similar reduction in ZO-1, intestinal permeability, steatosis, and fibrosis was observed in the choline-deficient/L-amino-acid-defined NASH diet, and was significantly attenuated by probiotic treatment with *Clostridium butyricum* MIYAIRI 588 strain. Interestingly, in mouse models of CCL_4_-induced fibrosis, different probiotics achieve improved intestinal permeability via unique mechanisms: *Bifidobacterium* probiotics have been shown to increase intestinal TJ expression [180] whereas *Lactobacillus-paracasei*-fermented milk reduces intestinal permeability by increasing antimicrobial β-defensin expression [181].

The study of prebiotics allows researchers to understand the relationship between a substrate, microbial metabolism, gut health, and permeability. The majority of prebiotics used today are indigestible carbohydrate polymers (fibers) that become fermented by gut bacteria to produce, among other things, short chain fatty acids (SCFAs) consisting of acetate, propionate, and butyrate [182]. SCFAs are significant homeostatic and anti-inflammatory signaling molecules in the gut (reviewed in Reference [183]), but can also significantly alter intestinal permeability. In vitro, butyrate has been shown to increase ZO-1 expression in Caco-2 cells [184], and stimulate mucous secretion in E12 human colon cells [185]. In vivo, rats supplemented with oats rich in fermentable β-glucans were significantly more resistant to alcohol-induced oxidative stress and intestinal permeability [186]. In humans, increased fiber intake has also been associated with improved permeability (reduced circulating ZO-1) and reduced ALT/AST in NALFD patients [187]. These data are supported by two recent meta-analyses finding that both prebiotics and probiotics reduce liver enzymes ALT, AST, and GGT in NAFLD patients [188], with probiotics also reducing serum ammonia and hepatic encephalopathy in cirrhotic patients [189].

### 4.2. Therapies to Dampen the Hepatic Immune Response

Direct inhibition of PRRs for the treatment of chronic liver disease has not been a research priority due to their peripheral contribution to disease pathogenesis. It is, however, now becoming evident that PRR activation from both microbial and self-ligands can significantly contribute to liver inflammation and fibrogenesis. TLR-agonistic therapies are currently being actively pursued for cancer therapies/adjuvants (reviewed in Reference [190]). TLR inhibitors, on the other hand, have been studied to a lesser degree in a handful of studies assessing their efficacy in inflammatory and autoimmune disease. Even fewer therapies are being assessed in the context of chronic liver disease, with TLR2/4/9, NLRP3, and STING pathway inhibition garnering some interest from pharmaceutical companies [190].

The humanized TLR2 mAb OPN-305 can achieve significant blockade of TLR2 inflammatory signaling in response to bacterial stimuli in healthy subjects [191], and improved overall response rate in patients with myelodysplastic syndromes that had previously failed hypomethylating agent therapy [192]. While TLR2 blockade has not been assessed in human CLD, the pleiotropic nature of TLR2 suggests that it may benefit inflammatory and fibrotic progression.

Small molecule inhibitors of TLR4, i.e., NI-0101 [193] and Ibudilast [194], are being assessed in extrahepatic disease, and JKB-121, a weak TLR4 antagonist, is currently being assessed in NASH [195]: preliminary results do not support a significant therapeutic benefit of JKB-121, though the study was confounded by a notable improvement in liver inflammation within the placebo group.

TLR9 blockade represents perhaps the most exciting TLR-targeting therapy. The TLR9 inhibitor hydroxychloroquine is currently used as treatment for autoimmune diseases such as rheumatoid arthritis and lupus, allowing potential repurposing for CLD. Furthermore, the novel TLR9 antagonist COV08-0064 has shown promise in animal models of sterile liver inflammation, particularly in the context of fatty liver where it limits inflammasome activation [196,197]. While TLR9 inhibition has yet to be assessed in human CLD, its diversity of microbial ligands, including bacterial, fungal, and phage DNA, will surely make it a candidate for future trials.

Research targeting the NLRP3–inflammasome pathway is undergoing rapid expansion alongside the number of inflammasome-associated diseases. While the NLRP3 inhibitor CP-456,773 was removed from phase II trials for rheumatoid arthritis due to supposed liver toxicity, other inhibitors are in different stages of development for the treatment of gout and Parkinson’s disease, among others [198]. In 2018, Genentec acquired San Diego-based Jecure Therapeutics and their portfolio of preclinical NLRP3 inhibitors as a treatment for NASH and hepatic fibrosis [199].

Lastly, the intracellular DNA sensor STING has become a popular target due to its association with autoimmune disease. STING inhibitors have been recently developed [200], and are being actively generated by a number of pharmaceutical companies with the aim of targeting STING-related genetic disease. This compound would have the potential to move towards therapies for CLDs such as NASH and fibrosis in the future [201].

## 5. Conclusions and Future Perspectives

In summary, this review has highlighted the strong connection between the liver and gut in the context of liver disease. Indeed, CLD does not occur in isolation, but is accompanied by disturbances of the complex balance of gastrointestinal microbiota, architecture, and immunity. Similarly, gut microbiota can play a significant role in liver health by altering host metabolism and immunity. While such associations are strong, it is much less certain whether the distinct disease states in CLD and fibrosis stimulate changes in microbiota, or if gut microbiota exacerbate inflammatory and fibrotic progression.

Host, microbiomic, and lifestyle factors that influence intestinal permeability are beginning to be understood, but there is still much to be learned. Specifically, there is poor understanding of the exact mechanisms by which intestinal permeability is altered and the physiological reasons for it. TJ expression and localization are often examined in isolation, while overlooking the numerous additional barriers that prevent microbial translocation in the gut. Antimicrobial peptides, IgA, and mucus abundance and localization are rarely examined in this context, and a better understanding of their regulation in health and disease is warranted.

**Figure 2:**
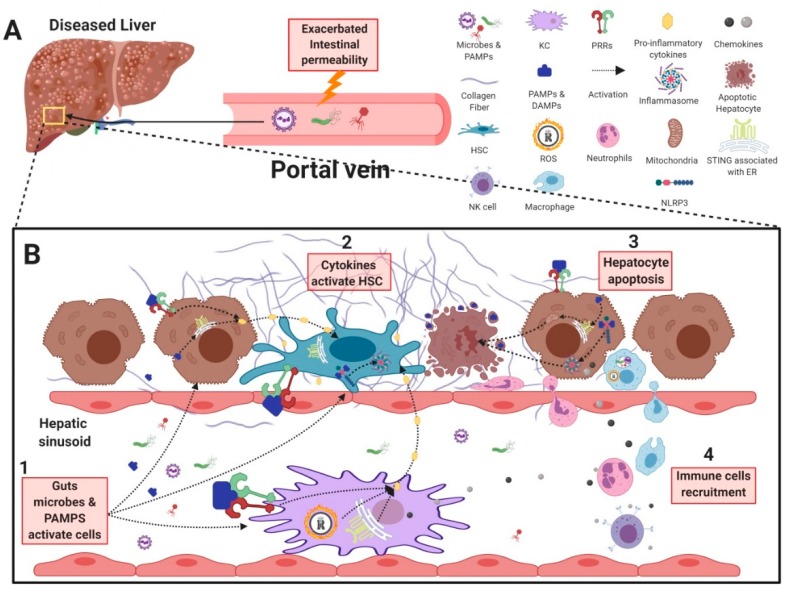
Gastrointestinal microbes and their contribution to liver inflammation and fibrosis. (**A**) In chronic liver disease, gut microbes and PAMPs can cross the intestinal barrier due to an increase in intestinal permeability, resulting in their transport into the liver through the hepatic portal vein. (**B1**) Gut-derived antigens are recognized by, and activate HSCs, KCs, and hepatocytes, resulting in the secretion of pro-inflammatory cytokines and chemokines. (**B2**) Pro-inflammatory cytokines produced from KCs and hepatocytes further activate HSCs to further exacerbate fibrogenesis. (**B3**) In addition, activation of STING– and NLRP3–inflammasome pathways in hepatocytes can trigger apoptosis and release of DAMPs to further activate HSCs. (**B4**) Chemokines produced by activated KCs, HSCs, and hepatocytes recruit immune cells such as neutrophils, NK cells, and monocytes to further exacerbate liver inflammation and injury. PAMP: pathogen associated molecular pattern; KC: Kupffer cell; HSC: hepatic stellate cell; NK cell: natural killer cell; PRR: pattern recognition receptor; TLR: Toll-like receptor; NOD: nucleotide-binding oligomerization domain-containing protein 1; NLRP3: nucleotide-binding oligomerization domain, leucine-rich repeat- and pyrin-domain-containing 3; STING: stimulator of interferon genes; DAMP: damage associate molecular pattern.

Lastly, along with public health initiatives aimed at reducing the causative lifestyle factors of fibrosis, i.e., alcohol and obesity, we must focus research on the development of novel PRR-antagonizing therapies. Murine studies have highlighted the contribution of individual PRRs to the development and progression of liver inflammation and fibrosis (as summarized in Figure 2); however, it remains unclear how multiple sensors collectively drive disease and may potentiate each signal. Human trials are pending to examine microbial sensors such as TLR9 and STING as well as inflammasome components to determine their contribution, whether alone or in concert, to fibrosis progression.

## Figures and Tables

**Table 1:** Intestinal barrier deficiencies in chronic liver disease.

Intestinal Barriers	ALD	NAFLD/NASH	Cirrhosis
**Mucus**	Reduced mucus production [52], fewer goblet cells [56,57]	N/A	N/A
**IgA**	Increased systemic IgA, reduced luminal IgA [63,64]	Reduced luminal IgA [75]	N/A
**Tight junctions**	Downregulated ZO-1, occludin, claudin [58,60], redistributed ZO-1 into cytoplasm [94]	Downregulated ZO-1 and occludin claudin switching [70,71,72]	Reduced occludin and claudin-1 [87]
**IECs**	Apoptosis [61]	N/A	N/A
**Gut immunity**	Mucosal-associated invariant T-cell depletion and impaired activation thereof [65]	Fewer IgA-positive cells [75], increased production of inflammatory cytokines (IFN-γ, IFN-α, IL-6) [76], increase in CD4^+^ and CD8^+^ T cells [76]	Release of inflammatory cytokines (TNF-α, IL-12) [90], increased DC sampling [91]

ALD: alcohol liver disease; NAFLD: non-alcoholic fatty liver disease; NASH: non-alcoholic steatohepatitis; TJ: tight junction; ZO: zonula occludens; IFN: interferon; IL: interleukin; CD: cluster of differentiation; TNF: tumor necrosis factor; DC: dendritic cell.

**Table 2:** Pattern recognition receptors (PRRs) that contribute to hepatic inflammation and fibrogenesis.

PRRs	Hepatic Cell Expression	Ligands	Human Disease Involvement	Mouse Model	Role in Hepatic Inflammation and Fibrogenesis
TLR2	Hepatocyte, KC, HSC [96,97]	β-glycan, zymosan, LPS, HMGB1	**↑** in NASH/NAFLD [98,99,100], **↓** in ALD [101].	**ALD**: Binge ethanol feeding	IL-1β, IL-6, and TNF-α **↑**, hepatic neutrophil **↑** [102,103].
				**NASH**: MCD diet	Protective effect against NASH and inflammation [104].
				**NASH**: CDAA diet	KC activation and proinflammatory cytokine **↑** Activation of HSCs to directly promote fibrosis [96].
				**Fibrosis**: BDL	No effect [105]
				**Fibrosis**: CCl_4_	CXCL2 **↑**, neutrophil **↑** [106]. HSC **↑**, α-SMA **↑** via MAPK and NF-κB pathways [107].
TLR3	Hepatocyte, KC, HSC, and LSEC [108,109,110,111,112]	dsRNA	N/A	**ALD**: HFD and binge drinking	IL-10 **↑** from KC and HSC, TNF-α, IL-2, CCL2 **↓** (anti-inflammatory) [113].
				**Fibrosis**: DDC/CCl_4_	HSC killing via NK cell activation (anti-fibrotic) [114].
				**Fibrosis**: CCl_4_	HSC activation, upregulated α-SMA, TGFβ, COL1A1 **↑** [115].
TLR4	Hepatocyte, KC, HSC, and LSEC [97,112]	LPS, HMGB1, and more	**↑** in NASH **↑** circulating LPS [100,116]. PBMCs from ALD are sensitized to LPS [117]. NAFLD-associated [118].	**ALD**: Lieber–deCarli diet	ROS **↑** inflammation **↑**, pro-inflammatory cytokine **↑** [119,120].
				**NASH**: HFHC-diet-fed ApoE KO	ROS **↑**, inflammatory cytokine **↑** from KCs and hepatic macrophages [121,122].
				**Fibrosis**: Bile duct ligation	TLR4–MyD88–NF-κB pathway triggered HSC activation, pro-inflammatory cytokine, pro-fibrotic gene **↑** [105,123].
				**Fibrosis**: TAK1 KO	Directly activated HSC, pro-inflammatory cytokine, pro-fibrotic gene **↑** [124].
TLR5	Hepatocyte, LSEC, HSC [97,112,125]	Flagellin, HMGB1	N/A	**Inflammation**: Flagellin injection	Inflammatory cytokine **↑**, macrophage and neutrophil recruitment [126].
				**NASH**: MCD diet	Hepatic inflammation **↓** inflammatory cytokines **↓**, deactivating HSC [125].
				**Fibrosis**: CCl_4_	Activated HSC via NF-κB and MAPK pathways to stimulate hepatic inflammation and collagen deposition [127].
TLR7	Hepatocyte, KC, and LSEC [112,128,129]	ssRNA	**↑** in ALD [130,131]	**ALD**: 25% (w/v) ethanol diet	Inflammatory cytokine, TLR7 agonist let-7b **↑** [131].
				**NASH**: MCD diet	TNF-α and IFN-α **↑** in KC and DC respectively to stimulate hepatic inflammation [132].
				**Fibrosis**: CCl_4_	Pro-inflammatory cytokine and pro-fibrotic gene **↑** [129].
TLR9	LSEC and KC [97]	Unmethylated CpG	**↑** in NASH [100,133]. **↑** circulating bacterial DNA in ALD, liver fibrosis and cirrhosis [134,135,136].	**NASH**: CDAA diet	**↑** IL-1β from KCs to **↑** hepatic inflammation. No direct fibrogenic effect [137,138].
				**ALD**: Chronic-binge ethanol feeding	IL-1β, CXCL1/2/5 **↑** from hepatocytes and HSC to recruit neutrophils [102,139].
				**Fibrosis**: BDL	Directly activated HSC [140].
				**Fibrosis**: Tak1ΔHep mice	Directly activated HSC [124].
NOD1	Hepatocyte, HSC, and KC [141,142].	LPS, flagellin, bacterial RNA, HMGB1, ATP	N/A	Mouse model of BDL/ CCl_4_-induced fibrosis	Recruited neutrophils to drive acute hepatic inflammation (CCl_4_ model) [143]. CXCL1, CCL5, inflammation, fibrosis **↑** (BDL/ CCl_4_ models).
NOD2				N/A	N/A
NLRP3				**NASH**: HFD	**↑** NLRP3 caused **↑** inflammatory cytokine [144,145].
Dectin-1	Hepatocyte and LSEC [146].	β-glucans	N/A	**ALD**: Lieber-DeCarli diet (4.5% ethanol v/v)	Plasma β-glucan **↑** drove **↑** KC inflammatory cytokine [147].
				**Fibrosis**: TAA/CCl_4_	Dectin-1 suppressed expression of TLR4 and CD14, inflammatory cytokine **↓** and activation of HSCs [146].
cGAS- cGAMP- STING	Hepatocyte, KC, HSC, and LSEC [112]	Cytosolic DNA and CDNs	STING **↑** in NAFLD [148]	**ALD**: Lieber–DeCarli diet (4.5% ethanol v/v)	STING–IRF3 pathway triggered hepatic pro-inflammatory cytokine production [149].
				**NASH**: HFD and MCD diet	mtDNA activated STING in KC [150].
				**Fibrosis**: CCl_4_	STING–IRF3 pathway activated hepatocyte apoptosis, HSC, and fibrogenesis [151].

PRR: pattern recognition receptor; TLR: Toll-like receptor; KC: Kupffer cell; HSC: hepatic stellate cell; LPS: lipopolysaccharide; HMGB1: high mobility group box 1 protein; NASH: non-alcoholic steatohepatitis; NAFLD: non-alcoholic fatty liver disease; ALD: alcoholic liver disease; TNF-α: tumor necrosis factor; MCD diet: methionine/choline-deficient diet; LSEC: liver sinusoidal endothelial cell; CDAA: choline-deficient L-amino-defined; CCl_4_: carbon tetrachloride; α-SMA: α-smooth muscle actin; MAPK: mitogen-activated protein kinase; NF-Κb: nuclear factor kappa-light-chain-enhancer of activated B cells; dsRNA: double-stranded RNA; HFD: highffat diet; DDC: 3,5-diethoxycarbonyl-1,4-dihydrocollidine; NK cell: natural killer cell; COL1A1: collagen type 1 A1; PBMC: peripheral blood mononuclear cell; ROS: reactive oxygen species; HFHC: high-fat, high-cholesterol; KO: knockout; TAK1: transforming growth factor beta-activated kinase 1; ssRNA: single-stranded RNA; IFN: interferon; DC: dendritic cell; NLR: NOD-like receptor; NOD: nucleotide-binding and oligomerization domain; NLRP: nucleotide-binding oligomerization domain, leucine-rich repeat- and pyrin-domain-containing; ATP: adenosine triphosphate; CLR: C-type lectin receptors; STING: Stimulator of Interferon Genes; cGAS: cyclic GMP-AMP Synthase; cGAMP: cyclic GMP-AMP; CDNs: cyclic dinucleotides; IRF3: interferon regulatory transcription factor 3; mtDNA: mitochondrial DNA. ↑: upregulation of expression; ↓: downregulation of expression.
